# The times and spaces of transplantation: queercrip histories as futurities

**DOI:** 10.1136/medhum-2021-012199

**Published:** 2021-08-20

**Authors:** Donna McCormack

**Affiliations:** School of Literature and Languages, University of Surrey, Guildford, UK

**Keywords:** queer theory, literature, immunology, literature and medicine, disability

## Abstract

With a focus on Larissa Lai’s *The Tiger Flu*, this article explores how transplantation is part of the ongoing transformation of being in a body that is of the world. That is, it examines how we may require other ways of thinking bodies as constituted by histories, spaces and times that may be ignored in the biomedical arena. *The Tiger Flu*, I argue, calls for an intra- and inter-connected way of thinking how we treat bodies, and thereby ways of working with bodies affected by environmental disasters (both acute and ongoing capitalist and colonial projects), multiple selves and time as more than linear. I turn to queercrip as a way of defying a curative imaginary that dominates transplantation and in so doing examine the colonial, capitalist violence of present day living. I move through Eve Hayward’s and Karen Barad’s work to examine how the cut of transplantation is a transformation, as integral to the ongoing experience of having a body in the world and yet potentially unique in its force of bringing inter- and intra-relatedness to the fore of one’s existence. Rather than sick or cured, I argue that transplantation is a transformation that captures our bodily changes, how the environment constitutes the self, how parts may feel integral to the self or easily disposed of, how viscera may tie us to others, and how the future may only be forged through a re-turn to the past (of the donor and a pre-transplant self). Transplantation is not about loss of self or gaining of an other, but rather about rendering apparent our multispecies, multiworld ties, and thus how we are bound by the histories we forge and the futures we re-member.

## Transplantation’s bodies: shifting temporal and spatial configurations

Transplantation’s histories, times and spaces are queercrip. That is, transplantation undoes the borders of Enlightenment Humanism by defying the supposed binary distinctions between death and life, self and other, and past and present (Haraway 2016; Hird 2006; Neimanis 2017). In this article, I argue that organs as spatiotemporal matter not only constitute the possibility of time and space for recipients—in the form of life extension and thus moving through the living world—but also carry histories that speculate on which futures and modes of belonging may be possible. I suggest that the transplanted body is always already conceptualised and experienced as a space insofar as it is a cavern into which the transplant team/surgeon cuts, excises the dying or dead organ(s), leaving a gap into which can be placed the donated organ. Similarly, the donor’s body is spatialised as that which can be entered and mined for its resources of organs. I use the mining metaphor to capture how organs may be seen as mobile matter that can be excavated for use by others, as well as to highlight how organ donation is intricately tied to ideas of capitalism, scarce resources and exploitation. This article argues that when the body is imagined as a container, time itself is always linear. Melinda Cooper insists that ‘the point of […] whole organ transplantation is to realize a seamless translation of the organ in space and time—point-to-point substitution—while suppressing any other kind of change’ (Cooper 2008, 110), and thus the aim is ‘to maintain the form and function of the organ in time’ (Cooper 2008, 123).1 I agree with Cooper that the body and its organs are conceptualised in organ transplantation as similarly identical parts, spaces and times. Form must broadly stay the same for the organ and the recipient. The temporality of the recipient must be restored so that life can then continue to move forward. The spatiality of transplantation is this body that contains parts that may be segregated, removed, replaced so as to reanimate an unchanged person in the same time and space. While there has been some work on time in transplantation (Haddow 2005; Kaufman 2010; McCormack 2015a,b; Shildrick 2019; Wasson 2020), there has been less on the role of space (Davies 2006). However, there is currently little on the relationship between time, space and transplantation, and this article aims to develop this conversation. Analysing the apocalyptic novel *The Tiger Flu*, this article parses out an antiracist queercrip spacetime approach to transplantation that troubles biomedical curative imaginaries of transplantation. This queercrip approach—informed by Indigenous, trans and posthumanist feminist theories—explores how the novel portrays and undoes colonial and late capitalist violence through the prism of transplantation. I argue that in this novel transplantation shifts from an individual intervention into a discrete body to an inter- and intra-action with environments and multi-beings. Thus, organ transplantation captures the non-linear, multispatial experiences of being in and of the world.

I begin by exploring the role of apocalypse in how we think the temporality of health and illness, with a specific focus on Indigenous formulations of apocalyptic narratives. While *The Tiger Flu* is not an Indigenous novel, its focus on a future imaginary space raises issues around land rights, biological destruction and repeated genocide that are redolent of Canada’s histories and its repeated violence to Indigenous peoples, migrants and those racialised as other. I lay out how a rethinking of the apocalypse as that which has already happened *and* that which is in process captures how apocalypses are not only about destruction, but also the potential to imagine other futures. In order to analyse how the novel portrays transplantation as a reimagining of alternative bodily forms and times, I draw out links between the traversing of geographical borders and the act of remembering. Crossing boundaries is an active undoing of racial segregation based on racist hierarchies, and thus becomes a way of not only eliminating this prejudicial violence, but also of forming alliances. I engage with queercrip to provide vocabulary for rethinking what takes place in the process of transplantation. My argument is simple: transplantation is part of the ongoing transformation of being in a body that is of the world. Yet, even while I want to emphasise its simplicity, I am arguing that we require other ways of thinking bodies as constituted by histories, spaces and times that may be ignored in the biomedical arena. *The Tiger Flu*, I suggest, calls for an intra- and inter-connected way of thinking how we treat bodies, and thereby ways of working with bodies affected by environmental disasters (both acute and ongoing capitalist and colonial projects), multiple selves and time as more than linear. Queercrip is a way of defying a curative imaginary (Kafer 2013) that dominates transplantation (Kierans 2005; McCormack 2021; Sharp 2006; Zeiler 2018) and in so doing re-turns to the colonial, capitalist violence of present-day living. I move through Eva Hayward’s and Karen Barad’s work to examine how the cut of transplantation is a transformation, integral to the ongoing experience of having a body in the world and yet potentially unique in its force of bringing inter- and intra-relatedness to the fore of one’s existence. I conclude by turning to fish and starfish, the latter being the figure of transplantation in the novel. These oceanic and queercrip creatures capture an imaginary of transplantation that transforms how we think the times, spaces and bodies of transplantation. Rather than sick or cured, I argue that transplantation is a transformation that captures our bodily changes, how the environment constitutes the self, how parts may feel integral to the self or easily disposed of, how viscera may tie us to others and how the future may only be forged through a re-turn to the past (of the donor and a pre-transplant self). Transplantation is one way of thinking how we are tied to others, and enfolded in time and space as that which makes and unmakes us and that which we un/make. Transformation is not about loss of self or gaining of an other, but rather about rendering apparent our multispecies, multiworld ties, and thus how we are bound by the histories we forge and the futures we re-member.

## Living apocalypses: environmental disasters, viruses and racism

*The Tiger Flu* was published prior to the global pandemic of COVID-19 and is therefore eerily prescient of what would emerge in December 2019. Particularly important is how COVID-19 came to be racialised as originating in Wuhan, China, and therefore presented by media and government sources as another *Asian contagion* (Chiu 2004; Singh, Davidson, and Borger 2020; Oliver 2011; Ty 2010). Lai has spoken about how *The Tiger Flu ‘*has roots in the Asian Avian Flu (H5N1) that began in Hong Kong in 2003 and arrived in Canada shortly afterwards’ (Cheng 2018), and has expressed how ‘the coverage of the flu in the mainstream press was so racist in its associat[ion] of Asian with contagion’ (Semel 2018). Viruses as racialised shows how health is structured through racism (Nelson 2011), as well as how illness constitutes the disposability of some populations and the lifesaving endeavours for others (Butler 2004). Lai raises the spectre of the AIDS crisis, where globally governments refused to respond (Diedrich 2007), and builds on this history of illness as impacting specific populations to examine what it would mean for a virus to largely only affect affluent men. In Lai’s words: ‘The historical precedent for a selective virus, is, of course AIDS, which favoured a number of oppressed populations. But what if a disease came that afflicted the abusive and affluent instead?’ (Cheng 2018). She continues: ‘And it got me thinking: “What if the flu were as specific in its biological targeting as the coverage of it was in its racial targeting?”’ (Semel 2018). Lai targets the biological as a category mobilised to define supposedly definitive difference, marking certain bodies as sick and inferior. Yet, she responds to the racist underpinnings of viral narratives (Wald 2008), which we are witnessing yet again during the COVID-19 pandemic, challenging these by having a flu that targets the most powerful in society, as if affluence and maleness are biological categories that viruses may attack. Lai undermines the racism mobilised as biological fact and thereby explores what would happen should those in power fall.

*The Tiger Flu* could be described as apocalyptic as the current world order collapses. While the virus tends only to affect rich, male adults, the rest of society still suffers greatly as food becomes scarce, borders start to close, money is elusive to most, and alternative forms of trade and labour rise. Indeed, the novel emphasises how viruses and pandemics exacerbate existing inequalities (Altschuler and Priscilla 2020). This apocalypse, as far as knowable origins are possible, is created by humans: the Caspian tiger was brought out of extinction by Kora Ko’s grandfather who extracted its DNA from a rug (Lai 2018, 85, 215, 226–27). Within this apocalyptic world of segregation through so-called ‘Quarantine Rings’, there are two women-only—but not so human—communities fighting for survival: the Cordova Dancing School for Girls, where Kora Ko is sent by her family, and the few surviving Grist sisters, cast out from Saltwater City into the Fourth Quarantine Ring, who must search for a new starfish if they are to survive as a community. Kirilow Groundsel leaves the Fourth Quarantine Ring, heads to Saltwater City and becomes the doctor at the Cordova Dancing School where she learns that the Grist sisters did not all leave Saltwater City. The starfish for whom she searches is the not-so-human-and-yet-human-looking being who can regrow body parts to donate to the doubler who brings into being new Grist sisters. *The Tiger Flu* is a speculative engagement with biotechnologies, histories of colonial racism, which include environmental disasters, alternative imaginaries of embodiment and belonging, endless forms of social violence and exclusion, and the future possibilities of late capitalism as it continues to destroy societies, communities and individuals.

As part of the ever-rising genre of speculative fiction, the novel forces us to address the question many Indigenous writers and writers of colour are asking: whose apocalypse are we discussing and, as Kim TallBear states, ‘I also think about the fact that as Indigenous people [we’re] already post-apocalyptic’ (TallBear 2017, 43:56). Indeed, Dallas Hunt, speaking specifically about apocalypses in the North American context, asks:

I do wonder if there is a generative way in which we can deploy the term apocalypse just in terms of thinking about the current configurations that structure our lives? […] What do we think about the apocalypse of settler colonialism? What would that then enable? (Hunt in Jackson et al. 2021, 27:50)

Here, Hunt reframes the apocalypse not as the end of times, which is particularly prominent in ecocriticism and Anthropocenic criticism (Carey 2019). Rather, the apocalypse is the downfall of the current power structures, of the existing colonial systems. The future is unknown and undoubtedly the impact will be felt across all communities, but we could ask with Hunt: is the end of these dominant, long-standing and extremely violent systems of power a future worth imagining? I would suggest that Lai’s novel explores Hunt’s questions. Indeed, Lai has questioned who is able to lay claims to the land in Canada, specifically interrogating Canada’s exclusionary migrant policy towards Asian populations:

Who ought to have the right to say who comes and goes? Those who were here first—aboriginal peoples? Those who planted a British flag on this soil? Or those who have been moving for centuries and continue to do so? Perhaps a little chaos is necessary in order to explore the answers to these questions. (Lai 2000, 40)

*The Tiger Flu* explores systemic inequalities as they relate to that which differentiates people. Characters are racialised as different through their geographic location, lines of segregation, histories of oppression, gender, health and how characters look. I therefore use Indigenous criticism here to examine how the novel poses questions about Canada’s nation state more generally, even while its focus is not specifically on Indigenous populations. I also read Lai’s novel as a reimagining of contemporary Canada that aligns with Hunt’s and TallBear’s arguments to give space to histories that are ignored, land claims that are yet to be acknowledged, violence that must stop and the possibility of futures for those repeatedly subject to state-supported genocides. The novel reveals the devastating impact the collapse of economic systems would have across the class spectrum, while also keeping to the fore the violence of these systems when they were in place. *The Tiger Flu* explores how characters are deeply impacted by the pre-apocalyptic system that was often lived as an apocalypse, and the current collapse of that system as bringing in another form of apocalypse that requires new ways of living be invented not by creating anew but by remembering what was and what could have been.

Memory is central to strategies of survival. Temporality is not only a forward-thrusting gesture to a future where human livability is triumphed as may be common in post-apocalyptic narratives. Here, the search for a future is only possible through a re-turn to the past. Remembering is the only way to continue on, and transplantation is key to this possibility. The Grist Sisters, a community of clones, expelled by their makers from Saltwater City ‘eighty years ago’ (Lai 2018, 20), survive through a doubler who releases eggs through parthenogenesis in this ‘lizardy love’ (Lai 2018, 20). The doubler births almost-clones who are from the ‘DNA of just one woman’ (Lai 2018, 20). In order to stay alive, the doubler requires a starfish to give her body parts, and the Grist community currently only has one starfish left, Peristrophe Halliana, which means when she dies (which she does) the community will also die. This is the plot line that propels the narrative as the groom (the lover and carer to the starfish) Kirilow Groundsel searches for a new starfish in Saltwater City, where it is slowly revealed some of the Grist community stayed in hiding. Organ transplantation drives Kirilow Groundsel to cross the borders of the city and to create connections with those she despises. The traversing of space is a crossing of times, as the past must be accessed in order to create a possible future. Lai has spoken about how she writes histories that have been silenced, particularly through racism (Lai 1999), and thereby creates novels that include ‘people who come from histories of travel and migration [and] people who are somehow marked as “other”, as different, whether that is through race, or a way of moving through space, or a way of speaking’ (Lai 2004, 170). The act of storytelling becomes the act of history making as we learn of the violence to the Grist sisters and the slow destruction of people’s lives across the various Quarantine Rings. *The Tiger Flu* addresses the environmental impact of apocalypses on the bodies of those within and ostracised from Saltwater City, tackling racism as an environmental issue not only as the bodies of the characters slowly deteriorate and die, but also as the multinational corporations take life with the promise of longevity. Isabelle Chow controls Saltwater City and the First Quarantine Ring, and is also the head of HöST, which offers freedom from the tiger flu by uploading one’s consciousness to the mainframe Eng and thereby being free of one’s body. The technology is not perfected yet, and thus Isabelle Chow’s promises are not only illusory, but also capture one of the main issues of the novel: that the separation of the mind from the body is central to the violence to which international, neoliberal corporations invite consumers to subject themselves. Indeed, at her most racist, Kirilow Groundsel points to the main issue with such technology: ‘This strange killing and rebirthing is Salty [racist term for someone from Saltwater City] business. We Grist sisters have no faith in such things. If the body is dead, then so is the woman, whatever these occultist Salties think they have copied’ (Lai 2018, 232).2 I would argue that this struggle between the capitalist body as separate from the mind, and thus the body as distinct from one’s self, is central to the reconceptualisation of transplantation that the novel offers. On the one hand, the text suggests that segregating people is a racist and violent act and is premised on a conceptualisation of the body as a discrete, isolated entity. On the other hand, it shows how existence is ‘human, animal and vegetable’ (Lai 2018, 186) and therefore that survival is an intra- and inter-connected collective mobilisation that seeks to reduce the violence of racialised segregation and to imagine otherwise. Indeed, the act of history making is, to go back to Hunt’s argument, a response to this apocalypse that seeks to build collectives in ways that do not mimic the violence of capitalism and its environmental destruction. Instead, this apocalypse is about how we imagine bodies and thus the body politic as intra- and inter-related with time, space (including the surrounding world) and other beings.

I would suggest that there is an alternative bodily formation in *The Tiger Flu* that is structured through and constitutive of non-linear temporalities and an active crossing of spatial boundaries. Apocalypse is a future projection, of that which ‘is yet to come’ (Murphy 2018, 117), and suggests an end of time as if apocalypses have not already been lived and survived. Yet, I follow Métis scholar Michelle Murphy’s questions: ‘For whom has massive violence not already been a daily struggle, and thus who has the luxury to think endangerments to life are in the future?’ (Murphy 2018, 117). The time of the already-lived apocalypse is the present of the neoliberal order where many profit from the violence to which the land and people are subjected. There is no ‘waiting for a better moment to arrive’ (Murphy 2018, 118). Time is to re-turn to memory to remember one’s relationalities, and such re-turns require the crossing of many forbidden and highly policed zones. Transplantation is key to this imaginary of embodied existence as enfolded in and by space and time, rather than a discrete body whose organs may be segregated from the space in which they reside and easily moved to another enclosed individual. Indeed, the Grist Sisters must undo the segregation to which they have been subjected by Isabelle Chow, defying spatialised separation and reuniting with aspects of their history they did not know and thereby accepting that they also are part of Saltwater City, despite their prejudiced views towards those they see as their oppressors: (the disparaging and racist term of) Salties. They build communities through the apocalypse in order to survive in a world order that actively negates their existence.

## Border crossings: segregation, linearity and queercrip

In this section, I argue for a queercrip reading of the novel and thus of transplantation. I use queercrip here because it forges a politics of the body that is as queer as it is crip. Queercrip, in the novel, captures how segregated communities, although isolated away, must still resist their ‘former masters’ (Lai 2018, 48). Invoking the ongoing power structures that imprisoned and now segregate the Grist sisters, Kirilow Groundsel’s mother double reminds her: ‘“The Grist may have evolved beyond its former masters, but we are not immune to their illnesses”’ (Lai 2018, 48). More specifically, queercrip captures how most of the girls suffer from environmental damage: they have data-like filigrees implanted into their skulls that get infected, bugs crawling round in their hair and of course the flu is a potential danger that girls can die from as we see from Peristrophe Halliana’s death (Lai 2018, 80). Kirilow Groundsel as the newly appointed doctor at the Cordova Dancing School for Girls describes what she sees:

The girls who live here are a dirty, thuggish lot. They have open sores on their faces that they try to cover with a flesh-colour liquid. […] They rim their eyes with charcoal pencils, which only accentuates the pus leaking from their tear ducts. Juicy, translucent lice play happy hopscotch in their matted hair. And infected metal threads drip off them like lace fungus from diseased trees. (Lai 2018, 160)

This queercrip perspective reveals ‘the gendered, laboring, and chronically toxically exposed bodies of globalized capital’ (Chen 2017, 130) that are often rendered non-apparent in the economisation of global life. Their ‘thuggish’ lives also manifest a potential resistance to the economic structure of daily life, although resistance is limited as they steal to survive. Environmental toxic, non-productive and repulsive monstrous bodies survive in dangerous and often deadly conditions.

I also use queercrip because transplantation is one of those illnesses that might not be defined or lived as a disability (Kafer 2013),3 and yet thinking transplantation as disability would account for the often long term illnesses that come prior to and after the transplant (Crowley-Matoka 2016). Indeed, I would suggest that speaking or thinking of transplantation as a cure4—a short-term intervention that will make everything better—covers over the complex ways in which illness returns, organs fail, transplants are repeated and new health issues occur. I use queercrip here for ‘the ethical, epistemic, and political responsibilities’ (Kafer 2013, 13) that come with the undoing of the binary of dis/abled, the reconfiguration of where bodily boundaries end, and how we account for the inter- and intra-dependence of being. Queercrip is a way of capturing how these bodies emerge through space and not simply how they live in an already made container. We are constituted by and constitutive of time and space: ‘I am not in space and time, nor do I conceive space and time; I belong to them, my body combines with them and includes them’ (Merleau-Ponty 1962, 161). While my focus is fiction, I am arguing that rethinking transplantation as cripqueer folds the body into time and space. One clear example of how this is already in play for organ transplantation is in the work of Sherine Hamdy (2012) who explores how kidney transplants in Egypt are often needed because of environmental pollutions and toxins: ‘the food and water that remade their blood [that of those people on dialysis] were polluted, just as the blood transfusions they needed might be contaminated’ (Hamdy 2012, 25). Transplantation is about the worlds we inhabit and how they form our bodies and about the inseparability of these, and thus the possibility of conceiving of a present and a future. Queercrip, here, is not an add-on to the queer temporality of thinking otherwise, but instead a way of interweaving the environmental and bodily into the times and spaces that the characters inhabit and forge in defiance of structural problems. It is about how transplantation exists within the world, in histories, spaces and times, and is thereby not an isolated body that waits for or donates separated organs. Bodies are intra- and inter-connected, and thereby we could rethink transplantation and other healthcare practices through these ties, particularly how one may feel a change in the self in the post-transplant context. While apocalyptic fictional worlds may appear far from biomedicine, I would suggest that the novel shows post-transplant existence as less about the normative, enclosed individual body and instead as potentially feeling ties to organs, to unknown dead people, to lost body parts, to an old self, to previously unimagined futures and histories, and more. In this configuration, bodies are always tied to others—founded through this relationality—and thus post-transplantation is less about how transplants alter embodied being and more about how it brings to the fore a biomedically ignored relationality. Importantly, time and space are not linear containers for the self, but shifting, mobile inter- and intra-relational visceral encounters. Binaries are the foundation for late capitalism and historical and ongoing forms of colonialism, and thus bodies as linear containers for organs are the figurative basis through which these systems of violence are maintained. Bearing witness to inter- and intra-species existence becomes one way of recognising one’s relationality with others and thus constituting less violent modes of being collectively on a damaged planet.

Furthermore, the queercrip formations of the starfish undo normative bodily structures, refuse a division between my survival and yours, and show that life is not a forward flowing force of healthiness but is instead damaged and destroyed by environments. In tandem with such destruction are multiple epistemologies and ontologies, including Kirilow Groundsel’s knowledge of foliage, fungi and other plant-based treatments; the Cordova Girls knowledge of ‘time before’ (Lai 2018, 31, 328) medications (ie, allopathic medicines that are rarely in circulation, such as ibuprofen); and the digital knowledge of Isabelle Chow, which allows chronically ill and dying people to upload their minds to one of the mainframes and thereby continue on living, which is redolent of right-to-die campaigns. As Isabelle Chow says:

[Men with the tiger flu are] going to die anyway, aren’t they? So we’re doing them a favour? You know this project is supposed to be a humanitarian one. […] You said they’d welcome Lïft, even if it isn’t perfect. It’s not perfect. I’d call it life-giving, yes, I believe it is. […] It’s better than dying, if you’re going to die anyway. (Lai 2018, 55)

There are many men who survive the tiger flu, chronically ill, living in male-only communities. Yet Isabelle Chow’s words point to the disposability of the life of those deemed unwell that is all too apparent in times of pandemics (Altschuler and Priscilla 2020; Chen 2020; Patton 2020). Disposability of bodies and their parts, as well as of environments (as constant replenishing sources that can be endlessly mined), is challenged by literal body and border crossing and thus delving into other histories. I am arguing that the movement across the Quarantine Rings, while a spatial act of traversing ‘clos[ed] borders to prevent infection in the plague rings’ (Lai 2018, 143), is also a temporal act of defiance and resistance. As one of the border police explains:

What I wouldn’t give to put a stop to these executions of innocent travellers. [What] these UMK [United Middle Kingdom] bastards call economic partnership, I call occupation, and with this tiger flu crisis, they just want to shoot everyone. If we could promise them the people could pass through *without remembering anything*, we could save some lives. (Lai 2018, 15 emphasis added)

Economic state and multinational corporate cooperation cover over the violence towards the population. Yet what allows Kirilow Groundsel and the Cordova girls to cross borders without being killed is not only one of the latter’s mothers being a lead figure in this legal cooperation, but also that they agree to forget by drinking the forget-me-do that the Grist sisters brew. Crossing borders is about remembering, as well as not allowing people to remember, and indeed the novel takes this up as its primary theme. As Kora Ko tells us at the end of the novel: ‘*But it was a time of information blackout. Everything they knew in the time before was stuck on Chang and Eng, and only the elites had access*’ (Lai 2018, 328).5 Knowledge and histories are locked away, accessible only to elites with economic power, and thus control of spatial borders is a reinforcement of a temporality that looks only forward. Should you look back—and this is also literally because if you look back while travelling across the border you will be shot—then death awaits. Inaccessible and deadly, knowledge and histories are isolated away to keep in place the Quarantine Rings that segregate peoples, and are forward-thrusting and thus constitute a temporality that looks only to a future of the same. The violence of constant border control as a public health policy designed to protect is as deadly as the virus itself; neoliberal government institutions require space to be segregated, populations to be available as biomatter and time to flow forward as if only the present is possible.

## Finding starfish: queercrip histories of futures

I want to turn specifically to the queercrip figure of the starfish, which along with the destruction of the Grist village ‘both here in the murderous present and there in the genocidal past’ (Lai 2018, 92), is the impetus for Kirilow Groundsel to head to Saltwater City, defying segregation rules to cross multiple borders to question the untrue and yet dominant history of the Grist sisters as the ‘captive mutants without honour, who all died in the first wave of tiger flu in the crowded scale factories of Saltwater City’ (Lai 2018, 89). To do this, I turn to Hayward’s ‘Lessons From a Starfish’ (Hayward 2008). I want to acknowledge that Hayward’s article is about ‘transsexual/transgender embodiment’ (Hayward 2008, 255) and that although my focus is not specifically trans bodies, I see this idea of the cut extending to organ transplantation where the continuation of life also depends on living with a transforming bodily form that takes place somewhere between the inside and the outside of the body and the self. I would also suggest that there is something queer about transplantation in its non-normative bodily and temporal forms (McCormack 2021). Hayward analyses the meaning of the prefixes *trans* and *re*:

*Trans-* prefix has more to do with the sense of across, through, over, to or on the other side of, beyond, outside of, from one place, person, thing or state to another. […] If we think about *re-* prefix however, the original sense of *re-* in Latin is that of ‘back’ or ‘backwards’, but in the numerous words formed by its usage, the prefix acquires various shades of meaning. For example, re-generate […]; and in biology, to replace (a lost or damaged organ or part) by the formation of new tissue. (Hayward 2008, 258)

Hayward insists trans identities and experiences are not solely about arrival into a desired fixed body, but instead a regenerative process that is always ongoing. I would add that thinking and living trans-as-ongoing-transformation-of-flesh-and-self captures how the prefix *re* is also about a turn to the past. For my argument here, re-turn, as well as re-place, are key in a number of ways: (1) there is the re-turn to the past where Kora Ko, who towards the end of the novel learns she is a starfish, must remember her history. As Kyra, a Cordova dancing girl, tells Kora Ko: ‘“In order to survive in the world that is coming, we need to know our history’” (Lai 2018, 86). The future is only possible through a constant re-turn to the past, and thus time is regenerative only in its disruptive, non-linear ways; (2) to re-place organs is to survive: that there be a starfish who can donate her organs to the doubler (who creates further Grist sisters) is about community continuity; and (3) where the trans of transplantation is about the movement of one (or more) organ(s) from one body to another, which may capture the illusion of restoration of wholeness in body and identity, this novel captures the ongoing ways in which the dead and histories live on in the ever-changing physical intra-activity of bodies, spaces and times. I take Barad’s conceptualisation of spacetimemattering and Lai’s starfish as a way of reading organ donation not as the revitalisation of an individual and separate life, but instead as the remembering of histories that cross boundaries of time, space and bodies. In other words, I use spacetime and spacetimematter to capture how the novel moves through queercrip bodies to reveal the constitutive ties of time, space and matter.

Hayward explicitly mentions transplantation and of course raises this issue because many echinoderms, such as the starfish, may purposefully lose their body parts as a defence mechanism or as a mode of recreating another starfish, and then reproduce another arm (Hayward 2008, 258). Indeed, this is precisely the role of the starfish in Lai’s novel, but she is more human in form, although they ‘aren’t human’ (Lai 2018, 48), and thereby can regrow body parts when these are removed to keep the doubler alive. The trans body, here, is cut, and there is not a re-turn to wholeness as this was not there originally. Indeed, Hayward argues that this is not about ‘hierarchies’ where one is whole and another fragmented (Hayward 2008, 256), but that ‘[we are] always of [our] tissue even in its ongoing transformation’ (Hayward 2008, 256). That is, regeneration could be described as a ‘re/iterative enactment of not only growing new boundaries (re-bodying), but of imperilling static boundaries (subjective transformation)’ (Hayward 2008, 258), ‘a phenomenological experience of re-shaping and re-working bodily boundaries’ (Hayward 2008, 259), and ‘the ways that we (starfish, transsexuals and others) autonomise and generate embodiment’ (Hayward 2008, 259). If I use *trans*, it is to capture the queercrip nature (where the culturenature separation is impossible when thinking of the starfish who undoes our human boundary epistemologies and yet cannot be anthropomorphised because of its bodily excess) of organ transplantation. It is not to conflate trans experiences with those who have undergone transplantation. Instead, it is to explore what might happen to the transplant experience if bodily boundaries were understood as influx, unstable, and the body as always in process. However, and importantly, is the impact this would have on the conceptions of time and space, where the goal of getting to somewhere—post-transplant healthiness—to resume linear time in a ‘body [as] container’ (Hayward 2008, 256) that moves through space would be transformed. Indeed, the celebratory tone of transplant narratives as one of survival is undermined in the novel as we witness the violence of the transplant process and that the starfish is given the forget-me-do drink to ensure she feels no pain, but also to keep her addicted to the donation process:

[The] first groom, inventor of the loving transplant, the sexy suture. […] Forget-me-do makes you feel pain as pleasure. It takes away all memory and feeling of pain, leaves nothing but a craving to be cut again. […] Peristrophe Halliana sighs a sleepy sigh of pleasure-pain. I move my fingers beneath her eyeball, the tiniest blade concealed between middle and index. Nudge it out and softly slice the root. She groans. I tug at the globe, and it releases with a gentle squelch and click. […] From her right eye, she gazes at me with love. (Lai 2018, 21–2)

In their segregated community, the Grist sisters survive through a violent process of constant forgetting in the form of organ transplantation. Isolated away, violence becomes indistinguishable from survival, and transplantation as a form of forgetting the past is integral to both survival and the perpetuation of violence. Indeed, Kirilow Groundsel as surgeon and groom demonstrates how love, violence and survival are impossible to separate from bodily cutting, sharing and regrowing.

When Hayward analyses the song ‘Cripple and the Starfish’ by Antony and the Johnsons (2002), she ignores the binary distinction of ‘life and death’ (Hayward 2008, 253) of which the song speaks and instead focuses solely on ‘male and female, human and animal’ (Hayward 2008, 253). I want to restore the centrality of this binary undoing to thinking the cut of transplantation as a possibility of living with transforming and regenerating bodily boundaries and parts. I want to situate this cut at the limit of life and death, as the possible undoing of where life does not end for the recipient (if the transplant is successful) and as a form of vitality for the deceased donor in the form of the transplanted organ. Thus life and death enfold time and space such that death is not the end of time but the start of another time in another being and transplantation is not the restoration of linear time (as if that were the case to begin with) but instead the beginning of a time where one’s transformed bodily structure is tied to histories of an other who one does not know (namely the dead donor). Post-transplant temporality is therefore a re-turn to the past of who one was and who this other was, while also living in the present as a transformed being who is carving out a space to continue on. Similarly, if western philosophy and some transplant teams may conceive of the body as a container, a space in which to segregate, remove and replace parts, post-transplant embodiment reinforces that the cut, the enfolding of an other into the self through the donated organ, is a transformation of how one feels about one’s body, as well as challenging one’s relationship with one’s self and others. This is a ‘being of the world in its dynamic specificity’ (Barad 2014, 229), where one is of a world that may be other to one’s self—in the sense of the donor being a separate person—but is transformed by the visceral encounter with this person’s donated parts. Thus, the world within which one moves becomes a world where one may live with parts of an other in the self such that parts are mobile, static, of others and in the self, and therefore an active environment that shapes the body’s sense of individuality, of connections and of being in the world as a temporal and spatial being. To quote Barad speaking of a different echinoderm, the brittlestar, described as a ‘cousin of the starfish’ (Barad 2014, 222):

The brittlestar’s bodily dynamism resists the familiar notion that space is a preexisting container, a stage on which actors take their places, and that time is the mere uniform ticking of a clock. Spacetime does not sit still while bodies are made and remade. The relationship of space, time, and matter is much more intimate. Matter does not move in space and time. Matter materializes and dynamically enfolds different spatialities and temporalities. […] Embodiment is a matter not of being specifically situated in the world but, rather, of being of the world in its dynamic specificity. (Barad 2014, 228-9)

The starfish, as an echinoderm, is, therefore, not simply a way of arguing that body parts may be ways to form relationality—in fact, if we follow Barad, she invites us to question the idea that connectivity requires ‘physical contiguity’ (Barad 2014, 230). Instead, the starfish allows us to explore how bodies are temporally and spatially created through change and thereby how the experience of time and space may be impacted. The queercrip of transplantation is how normative notions of time and space may be challenged through the experience of transplantation, but also how the body itself becomes not a container for organs and instead a changing physical experience of being in the world and thus is not necessarily oriented by notions of healthiness and sickness. In this sense, transplantation is as environmental as it is biomedical, as visceral as it is metaphysical, as queer as it is crip. If organs move as if bodies are static spaces held in time that will be restored to normative healthiness, then transplantation belies these fixed ideas as organs moves across space and time, bringing together multiple temporalities and not restoring wholeness but rather bringing more questions about bodily relationality, about life and death, about where I end and you begin, about how we remember the dead, and so many more uncertainties about being of and with the world. The latter may be distressing, but it is also the potential to live with the uncertain changes that emerge as one’s body ails, is supposedly repaired through transplantation, and the many different senses of being in a transforming body that may constitute very different times and spaces that are tied to unknown pasts (of the donor) and unpredictable futures (of the recipient and their worlds).

Kinship may be forged through transplantation (Sharp 2006) almost in the repetition of literal blood (or matter) ties. However, in this novel, I want to suggest that a different form of kinship emerges through the search for a new starfish to save the Grist community after they have been subjected to another genocide by Höst. Through the search for a new starfish the novel creates modes of kinship that ‘are working hard to activate decolonial potentials now, without waiting for a better moment to arrive’ (Murphy 2018, 118) and in so doing that reconfigure—without necessarily eliminating—‘capitalism, colonialism, the nation-state or heteropatriarchy as world orders’ (Murphy 2018, 106). The prefix *re*, that Hayward speaks of, is a cut of the starfish: Kirilow purposefully cuts off Kora Ko’s hand to save her from dying of blood poisoning and to find out if she is a starfish. Indeed, her lifesaving measures are impossible to separate from her desperate search for the being who will save her community:

When I check again, there are blood blisters all over the hand. It could get better, but it’s not likely. A wicked thought crosses my mind. What if she is like the Salty that came through our woods? What if she is like Madame’s lost Carmela Sweetwater? Her hair is black like that of the Fourth Plague Ring Grist sisters, not red like the Cordova School ones. More than any other Salty I’ve met so far, she looks like she might be related to us. In any case I have a duty to stop the gangrene from eating up more than the hand. (Lai 2018, 185)

Bodies are chopped up, parts cut off, to regenerate community as Kirilow Groundsel suspects Kora Ko looks like the Grist Sisters and is thereby related although different. In an act that is as selfish as it is potentially curative, Kirilow Groundsel enacts violence on many levels: she amputates without consent and does so partly for her own future, and continues to denigrate the Saltwater City population with her racist use of ‘Salty’. At the same time, there is a queercripping of this curative imaginary (Kafer 2013) as the novel unfolds bodies in a temporal and spatial plain of ongoing environmental damage, where this refers to both the impact of ongoing industrial endeavours which result in pandemics and survival in this violent world. In other words, as Murphy argues, there is ‘a sense of relational living-being that extends not only outward into multi-species and land relations, but out into the very physical infrastructures of capitalism, colonialism, and racism. Or put another way, it offers a sense of how such infrastructures are physically present inside of us, unevenly distributing harms and supports’ (Murphy 2018, 115–6). The curative imaginary relies on a narrative of the future ‘that positions a medicalized cure as just around the corner, as arriving any minute now’ (Kafer 2013, 44). Yet I would suggest that what takes place here is queercrip insofar as Kirilow Groundsel is trying to forge kinship with a person who represents a group of people she hates because they forced her ancestors out of the city and in that it projects into the future the lives of those who should not live, those who should die from environmental toxicity, little access to healthcare and violence from the city leaders who own the technology that could kill the population. A future, where the Cordova Dancing Girls survive, is projected in the novel as a queercrip vision where bodies are changing, permeable, short-lived, unstable and unpredictable (Kafer 2013, 41). This is not a heteronormative vision of the nation state where disabled bodies are disposable, but a queercrip selfish and yet selfless act to save one supposedly unworthy life and to explore potential kin ties with those violent towards the Grist sisters’ ancestors. Neither Kirilow Groundsel nor Kora Ko are destined to survive in this violent, divisive context, and yet they are bumpily mapping out spaces into the future.

The reconfiguring of Kora Ko’s body, much to the horror of Kora Ko, is, I would argue, what Rosemarie Garland-Thomson describes as ‘[k]nowledge emerg[ing] in the form of differing bodyminds moving through environments together, navigating barriers, and finding pathways, both materially and metaphorically’ (Garland-Thompson in McRuer and Johnson 2014, 154). The Cordova Dancing girls are dependent on Kirilow Groundsel’s knowledge of plant- and herb-based medicine to survive, and yet her occupying a space in the girls’ house folds time back to when the Grist sisters were banished from Saltwater City, as well as towards a future where a starfish may live and thus the community survive. Acts of survival—leaving the Grist community in the Fourth Plague Ring, forced movement for Kora Ko out of the family home, seeking medical attention and amputating Kora’s hand—are timespacematterings. That is, while they are in and of space and time, they also constitute time and space by projecting queercrip life into the future, by creating a space of potential survival and by living—and having no choice about—their inter-connectedness, which is also an intra-connectedness that goes into their DNA of the past, present and future. It is as Murphy suggests that ‘*the very premise of the discrete body is unravelling*. [The] environments of our ancestors may be present inside us as *inherited metabolic patterns*’ (Murphy 2018, 115–6). The destructive environment that harms the mindbodies of these characters in its capitalist, racist and colonial structures cannot contain all epistemologies and ontologies. Indeed, as David Serlin argues, ‘many ways of being and knowing, unbeing and unknowing, exceed and make detours around capital or get stuck in-between economies, identities, and desires’ (Serlin in McRuer and Johnson 2014: 152). Kora Ko and Kirilow Groundsel start to unmake and unknow and in so doing must re-turn to the past to propel a futurity for these queercrips. The cut, as Hayward argues, becomes transformative and regenerative as a metaphor for ongoing vitality and renewal and literally as body parts multiply in non/a-human and echinoderm ways. The search for a starfish, the donor who will help the Grist sisters survive in spite of the social destruction, is the cut that opens the body to another future. This body has lived with acid rain (Lai 2018, 26), food lacking in nutrients (Lai 2018, 23), visible bugs living on her skull and now a ‘monster hand’ (Lai 2018, 221). This is ‘slow death’ (Berlant 2007) by institutional means, but Kora Ko’s moving to the Cordova Dancing School is a lesson in history, one she must learn in order to understand her family’s role in the flu, and an opening to another mode of living, which is, as Serlin suggests, a detour or being in-between systems, bodies, times and spaces (Serlin in McRuer and Johnson 2014, 152), or, as Murphy says, ‘making life otherwise in the ongoing fallout’ (Murphy 2018, 117). Transplantation as a means of survival—the cut as the potential for transformation—is the act that brings these disparate beings together. Transplantation as a cut into the individual body as a discrete space filled with individual organs is not what happens here. Instead, it is an act of queercrip projection into the future where the community may survive against the will of those in charge (Isabelle Chow) and where bodily undoings, intra-relatedness, environmental entanglements become the basis for revisioning belonging. All this takes place through the visceral acts of re-membering.

## Narrating histories: the past as future, ocean critters and trees of organs

Stinky, dirty girls are central to Lai’s fiction, and fish is a key feature of these worlds. Lai's previous novel *Salt Fish Girl* (Lai 2002) is a multi-origins story of humans emerging from the sea and humans who smell in a disease-like, repulsive way that is a means of remembering. Beings and foods—fish and durians, for example—are smelly motifs that evoke connotations of Asianness, foreignness, disgust and women-as-repulsive (Lai 2008; Oliver 2011; Ty 2010; Wong 2003). In *The Tiger Flu* Isabelle Chow needs the DNA from the Grist sisters for the success of her profit-making, future-promising, endless life business. In a largely unexplained process, the Grist sisters are put in the new technology Lïft and transformed into fish. Lai shows how profits depend on destroying lives, such that killing a whole population is essential to make money, and thus that the apocalypse is now in this space, and not in the future. Furthermore, unbeknownst to Kirilow Groundsel and Kora Ko when they are imprisoned, they are fed fish, which are their sisters. The technology used to extract DNA from the sisters nourishes the unsuspecting incarcerated characters, turning survival into unwitting consumption of the bodies of others, including one’s own community. Lai reveals the dangers of transnational neoliberalism where it is impossible not to consume the violence of multinational conglomerates. Death—as dead fish and thereby murdered Grist sisters—results in sustenance for these prisoners, while revealing the violence of biotechnologies of life, of extending life. Biotechnological life, where one’s consciousness will be extracted from the body and sent to the mainframe Eng, is inseparable from the genocide of the Grist sisters. Indeed, to re-turn to Hayward, the binary between life and death is transformed as companies sell the endless deferral of death to their customers, the desire for eternal life as a reality that reinforces some must die for others, that there are lesser humans, non-humans and ahumans who can die for human survival. Apocalypse in the current capitalist system is the survival of humans at any cost, as a separation of mind from body, where the self resides in a digital, non-visceral consciousness.

However, I would argue that the novel resists such structures of the self, and uses transplantation as the mode through which to do this. Fish as smelly and the starfish as the donor to ensure community survival situate transplantation on a planetary scale where donation and life extension requiring organs is no longer about individual diagnoses as such. The final chapter of the novel, which takes place 156 years after the previous chapter and section, reveals the queercrip temporality of these endangered lives that survive through a non/in/a-human (Giffney and Hird 2008; Luciana and Chen 2015), fish and tree time and space. Entitled ‘The Starfish Tree’, this short concluding chapter focuses on the Kora Tree, who has ‘no mouth’ but ‘vibrates language’ (Lai 2018, 327), telling the following to the ‘*litter*’ (Lai 2018, 327) of ‘young daughter doubles’ (Lai 2018, 328):

When you pluck your first replacement heart or liver from my branches, don’t you dare scoff. You must remember my pain, as I remember yours [of birthing]. […] I nearly died. I had to be uploaded to a batterkite and become its consciousness. And then we discovered that the tentacles of the kite doctored carefully and left to lie long enough atop a fertile soil could become roots. (Lai 2018, 327–328)

In a final twist of survival, Kora Ko is put in the Lïft—the technology designed to kill her—and transformed into a ‘giant fish […], silver and slippery, covered in long tendrilly scales, with a pink right fin’ (Lai 2018, 322). She becomes a fish, a smelly creature that evokes histories of migration and racism, while also reoccurring in Lai’s work as a sensorial memory, a memory carried in the smell (Lai 2008; Oliver 2011). The ‘pink right fin’ is the regeneration of her amputated hand-now-fin as she is transformed from human to fish. Community survival is not equivalent to the existence of humans. The Grist already walk the line of having been created in a factory and yet human presenting, but now their source of organs is a human turned fish transported in ‘a genetically modified flying squid whose primary purpose is as a military abduction and destruction unit’ (Beard 2019, 82) which is then planted on the soil to grow downwards and upwards in the form of a vibrating tree named the Kora Tree. If a tree of organs is redolent of the racist, colonial structure of neo-Darwinism and of a capitalist-driven market for endless supplies of replaceable body parts, the Kora Tree exceeds such violent structures of exclusion. Alive as a communicating vibration, the Kora Tree narrates histories, as well as providing essential organs to keep the now flourishing Grist sisters alive. The Kora Tree reaches down and up and back and forth projecting the genocide of communities into survival in the present. If I translated such speculatively fictionalised representations of transplantation into our everyday experiences of life-extending biotechnologies, I would emphasise that the current biomedical model constitutes and reinforces the body and its parts as isolated components distinct from their environments (and within this I include bodily ecologies). Such practices ignore how bodies are forged through the worlds in which we exist and thus how cutting our bodies changes how we experience time, space and self/other relationality. Thinking viscera as integral to who we are and histories as constitutive of self/other, space and time challenges transplant practices to forge the present as not solely curative but also queercrip where we live with chronic illness, disability and attachments to all sorts of queercrip ecologies (Taylor 2019).6

Kora’s survival as not-self and yet-still-self, as donor of organs and of histories, is a story of transplant survival, but not as a linear temporality of healthiness where organs in containers are replaced from another discrete, individual body. Instead, Kora’s life is a spatiotemporal transformative and regenerative process not as self but as something other than self within the self, or we might argue as a way of imagining how we live with all these lives in ourselves: the lives of ancestors (to re-turn to Murphy), donors, multiple beings already in the supposed self and the so-called environment in its intra-active processes. As Hayward and Barad argue, bodies are not containers and body parts transform who we are in fundamental and sometimes insignificant ways, as well as how we move through and are moved by time and space. Kora Ko is transformed at the moment of death—by yet another genocide on the Grist sisters—into a fish by a technology, the Lïft, designed to kill the Grist sisters. Genocides are the everyday acts of violence of neoliberal institutions that invent new ways of life that appeal to so many and yet are dependent on apocalypses in the now. This is where I would suggest Indigenous theorising opens up readings of apocalypses as not the end of the world, but the ‘destruction of particular life worlds’ (Hunt in Jackson et al. 2021, 17:10), which following Hunt’s formulation is the violence to which Indigenous populations—and in Lai’s text to which those who are racialised as other—are subjected. Yet Hunt insists this is also potentiality: ‘Just because these apocalypses have been attempted does not mean that they were successful. […] It is precisely because they were not that […] we envision the future, project into the future’ (Hunt in Jackson et al. 2021, 17:46). Indeed, Piepzna-Samarasinha (2020) suggests that disability justice is a ‘cripping of the apocalypse’ because ‘we’ve [disabled queer and trans Black, Indigenous and people of colour] already survived the end of the world’ (Piepzna-Samarasinha 2020, 133). Indigenous and queercrip formulations by writers of colour make apparent how apocalypses have been and continue to be survived. Lai’s novel is a queercrip call to listen to these apocalyptic experiences as histories of Indigenous peoples, people of colour, queers and the disabled, as well as of future imaginings. Multispecies transformations are queercrip modes of witnessing how bodies, lives and consciousnesses transform within what we might call one life. Some bodily changes—such as transplantation—may change one’s sense of self, time and space. If organs feel like separate entities that can be segregated away just as the Grist sisters were, then here they emerge from a multispecies intra-action: resistance against a militaristic genocide and an attempt to live otherwise in a queercrip intra-active mode of being with plants, the sea and one’s chosen family. This fantastical ending queercrips transplantation as that which brings to the fore how bodies, selves, times, spaces and being-in-the-world are transformed by biotechnologies. Our bodies are of the world, and thus becoming fish in a jellyfish that becomes a tree is one way of capturing how bodies are transformed by genocides, biotechnologies, histories and the possibility of survival. In other words, it is to ask TallBear’s temporally distorted question: ‘What have we learned in the apocalypse that we’ve already been living through now?’ (TallBear 2017, 45:04). Now is the time for the recognition of the historical and ongoing apocalypses that Indigenous peoples and all those othered in a myriad of ways have survived, and thereby to remember such strategies to create a present that is forged through histories of violence and survival against colonialism, capitalism, life-extending but often deadly biotechnologies and collective amnesia. As the Kora Tree reminds us: ‘*You must remember my pain, as I remember yours’* (Lai 2018, 327). There is no further mention of transplantation as requiring forget-me-do that keeps the starfish addicted to the pain-as-pleasure of the cut. Instead, we must re-member. Transplantation is about re-membering how bodies are enfolded by time and space, and thus that transformations to that body could bring about immense changes or realisations that one’s body is multiple, one’s histories are inter-related, bodies constitute and are formed by space and thus that bodies are viscerally intra- and inter-connected, and thereby that time moves in multiple and often unpredictable ways. Trans-as-bodies-constantly-changing bears witness to the histories of donors that may not be known in their entirety (or at all), to the idea of living with dead others in the self, and to feeling tied to spaces and times of an other. Queercrip transplants are spacetimematterings where the sensorial register and viscera may bring forth alternative histories, times and spaces.

## Data Availability

There are no data in this work.

## Notes

1. Even while techniques of transplantation are in flux and on some levels have changed since Cooper published her work, the conceptualisation of organ transplantation, I would suggest, is the same: maintain the shape of the body, reinsert a similar shape in the form of the same organ and restore chronological time.

2. Lai makes Kirilow Groundsel’s racism apparent throughout the novel to highlight how histories of oppression fuel racism on all sides. However, the novel is clear that the impact of such racism is not the same for those with access to resources, technologies, healthcare, and ultimately money and power.

3. Kafer does not include transplantation, but I think transplantation is an excellent example of where one might identify or actively shun the category of disability, qualify for the state status of disability in some countries or not depending on one’s health and the various conditions that come prior to and with transplantation.

4. One example of this is from the Scottish NHS website, which describes life after a liver transplant in the following way: ‘Recovering from a liver transplant can take a long time, but most people will gradually return to many of their normal activities within a few months’ (NHSinform 2021). While they do not use the term ‘cure’, NHS Scotland ignores the many illnesses that emerge after a liver transplant. There is a ‘Complications’ page which describes some post-transplant illnesses, but nowhere is there an indication that post-transplant life may be a disabled or chronically ill one.

5. Although I do not address it here, there is a further link to the reimagining of embodiment and defying racist, capitalist norms through the off-world sites Chang and Eng. This is a clear reference to the famous Asian conjoined twins, who were never separated, were displayed in freak shows and married. As many have discussed, conjoined twins defy biomedical norms of human embodiment where they are treated as two distinct people who must be separated, regardless of what conjoined twins or their parents want. See, for example, Garland Thomson 1996 and Fielder 1981.

6. I build on Sunaura Taylor’s ‘Disabled Ecologies’, which does not engage ‘queer’, to develop how transplant lives are queercrip in their myriad temporalities, spatialities and visceral forms.
